# Novel variant in *HHAT* as a cause of different sex development with partial gonadal dysgenesis associated with microcephaly, eye defects, and distal phalangeal hypoplasia of both thumbs: Case report

**DOI:** 10.3389/fendo.2022.957969

**Published:** 2022-10-11

**Authors:** Noelia Baz-Redón, Laura Soler-Colomer, Mónica Fernández-Cancio, Sara Benito-Sanz, Marta Garrido, Teresa Moliné, María Clemente, Núria Camats-Tarruella, Diego Yeste

**Affiliations:** ^1^ Growth and Development Group, Vall d’Hebron Research Institute (VHIR), Hospital Universitari Vall d’Hebron, Barcelona, Spain; ^2^ Pediatrics, Obstetrics and Gynecology and Preventive Medicine Department, Universitat Autònoma de Barcelona, Barcelona, Spain; ^3^ Pediatric Endocrinology Section, Hospital Universitari Vall d’Hebron, Barcelona, Spain; ^4^ Centre for Biomedical Network Research on Rare Diseases (CIBERER), Instituto de Salud Carlos III (ISCIII), Madrid, Spain; ^5^ Institute of Medical and Molecular Genetics (INGEMM), Hospital Universitario La Paz, Universidad Autonóma de Madrid, Madrid, Spain; ^6^ Department of Pathology, Hospital Universitari Vall d’Hebron, Barcelona, Spain

**Keywords:** 46,XY different sexual development, syndromic DSD, HHAT, minigene studies, case report

## Abstract

The palmitoylation of the Hedgehog (Hh) family of morphogens, named sonic hedgehog (SHH), desert hedgehog (DHH), and Indian hedgehog (IHH), is crucial for effective short- and long-range signaling. The hedgehog acyltransferase (HHAT) attaches the palmitate molecule to the Hh; therefore, variants in *HHAT* cause a broad spectrum of phenotypes. A missense *HHAT* novel variant c.1001T>A/p.(Met334Lys) was described in a patient first referred for a 46,XY different sexual development with partial gonadal dysgenesis but with microcephaly, eye defects, and distal phalangeal hypoplasia of both thumbs. The *in silico* analysis of the variant predicted an affectation of the nearest splicing site. Thus, *in vitro* minigene studies were carried out, which demonstrated that the variant does not affect the splicing. Subsequent protein *in silico* studies supported the pathogenicity of the variant, and, in conclusion, this was considered the cause of the patient’s phenotype.

## Introduction

**Figure 1:**
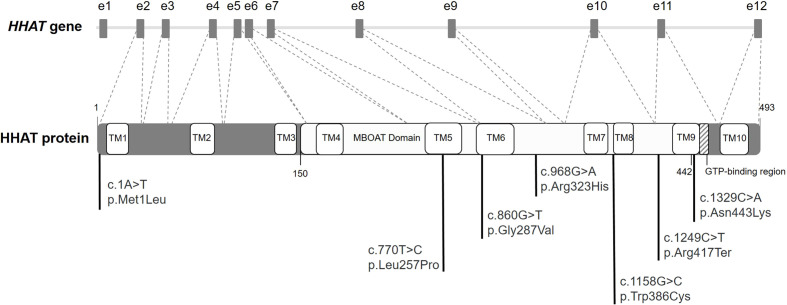
Diagram of *HHAT* gene and protein. Diagram of *HHAT* gene (NC_000001.11) with the boxes indicating the 12 exons (e1 to e12). The coding DNA sequence (NM_018194) from e2 to e12 codifies for HHAT protein (striped lines). Diagram of the HHAT protein isoform NP_060664.2 with its different domains and localization of the previously described variants in literature. The membrane-bound *O-*acyltransferase (MBOAT) domain encompasses amino acids 150 to 442.

The Hedgehog (Hh) family consists of morphogens implicated in the control of cell growth, survival, differentiation, and embryonic pattern for a variety of organs and tissues (1). The three Hh ligands are sonic hedgehog (SHH), desert hedgehog (DHH), and Indian hedgehog (IHH) (1). SHH is expressed in early embryogenesis and acts in neural, cranial, skeletal, and limb patterning. Later in development, during organogenesis, SHH is expressed in most epithelial tissues and is involved in the development of lungs, kidneys, hair, teeth, and eyes (1). Loss of SHH expression and function causes cyclopia and defects in the patterning of the ventral neural tube, somites, and foregut. Also, later defects include severe distal limb malformation, absence of vertebrae and most of the ribs, and failure in lung branching (1). DHH expression is largely restricted to gonads, so it is crucial for testis and ovarian development and function (1, 2). It is secreted by Sertoli cells and induces the formation of the Leydig cell lineage (3). Genetic variants in *DHH* cause 46,XY different sexual development (DSD) with partial or complete gonadal dysgenesis (2–6). IHH is specifically expressed in a limited number of tissues and acts on bone growth and pancreas development. Defects in *IHH* gene reduce chondrocyte proliferation causing bone growth defects such as dwarfism, acrocapitofemoral dysplasia, or brachydactyly (1, 7). Hh proteins undergo multiple processing steps, during their passage through the secretory pathway, required for the generation and release of the active functional ligand. After the signal sequence is removed, the Hh proteins suffer an autoproteolysis followed by a linkage of a cholesterol molecule to the C-terminus. Subsequently, a molecule of palmitate is covalently attached to the N-terminus of Hh by the hedgehog acyltransferase (HHAT). HHAT is a protein with 10 transmembrane domains and a catalytic membrane-bound *O-*acyltransferase (MBOAT) domain (amino acids 150–442) (**Figure 1**) (1, 8, 9). This palmitoylation is critical for effective short- and long-range signaling of Hh molecules (8).

*HHAT* gene (MIM: 605743) contains 12 exons, with its coding DNA sequence (CDS) extending from exon 2 to 12. Genetic variants in this gene have been related to Nivelon–Nivelon–Mabille syndrome with phenotypes similar to the loss of Hh function (**Figure 1**) (10). To date, and according to our knowledge, a total of 10 *HHAT* variants have been described in the literature, six of them missense, three gross deletions, and one nonsense. The described deletions were associated with schizophrenia (11), ankylosing spondylitis (12), and persistent cloaca (13). Regarding the nonsense variant, it was described in a case with pituitary stalk interruption syndrome (PSIS) (14). Conversely, the missense variants were reported in patients with a broad spectrum of phenotypes according to the impairment of SHH (i.e., intellectual disability, eye abnormalities, or limb malformations), DHH (DSD), and/or IHH (i.e., dwarfism or brachydactyly) (**Figure 1**) (7, 15–19).

We present an interesting case of a patient with a *HHAT* novel variant first referred for DSD but with a later diagnosis of affectation in the other Hh pathways. Moreover, the candidate *HHAT* variant was an exonic variant with a predicted splicing effect, so it was studied *in vitro*.

## Case report

The patient is a 13-year-old Pakistani girl who was admitted to the pediatric endocrinology outpatient clinic for hirsutism and lack of breast development. She was born by normal vaginal delivery at 38 weeks of gestation with a birth weight of 2,025 g (−2.5 SDS for gestational age) after an uneventful pregnancy. Her parents were first cousins, and she has two younger unaffected female siblings. No remarkable familial medical history was reported. Due to microcephaly, a brain magnetic resonance imaging (MRI), performed at the age of 7, showed biparietal gliosis and necrosis caused by a hypoxic–ischemic perinatal injury. She does not suffer from an intellectual disability. When she was almost 8 years old, she was diagnosed with different sexual development, with hypoplastic majora and minora labia, and a microphallus (length and width unavailable). No other clinical signs of hyperandrogenism were detected. Her external genital score (EGS) was 0 according to van der Straaten et al. (20) and Ahmed and Rodie (21).

Laboratory hormonal assays revealed high total testosterone levels of 20 ng/dl (normal reference range for prepubertal female <10 ng/dl) and normal levels of 17-hydroxyprogesterone at 0.52 ng/ml (normal reference range <1 ng/ml).

Physical examination showed that the patient had external genitalia virilization, with an increased amount of pubic hair extended to her inner thigh area, microphallus, and hypoplastic labia. Her urethral meatus was located on the ventral side of the phallus. She had no palpable gonads, and a vaginal introitus was present. Her shoulders were relatively broad, and no breast development was observed. Her voice was deepened, and her hirsutism, graded with a modified Ferriman and Gallwey score, was severe. The patient’s weight was 42.5 kg (−0.8 SDS), and her height was 162.8 cm (+1 SDS). She had no abnormal dysmorphic features.

Laboratory assays revealed the following hormonal values: luteinizing hormone (LH) 23.9 IU/L, follicle-stimulating hormone (FSH) 103.5 IU/L, estradiol 25.5 pg/ml, testosterone 325.6 ng/ml, dihydrotestosterone 280 pg/ml, androstenedione 2.2 ng/ml, dehydroepiandrosterone sulfate (DHEAS) 181.48 μg/dl, 17-hydroxyprogesterone 1.26 ng/ml, cortisol 22.8 μg/dl, anti-Müllerian hormone (AMH) 0.45 ng/ml, inhibin B 21.6 pg/ml, and prolactin 32.3 ng/ml. Her karyotype was 46,XY. The abdominopelvic ultrasound showed the absence of a uterus and ovaries, a filiform vagina up to its lower third, and two oval-shaped structures located in the upper third of the inguinal canal, probably corresponding to degenerated testes. These structures presented microcalcifications and vascularization, and the one on the left side had millimetric hypoechoic lesions, which can be considered suspicious for malignancy. A pelvic MRI confirmed these findings, showing bilateral vas deferens and seminal vesicles. Tumor markers (α-fetoprotein (AFP), human chorionic gonadotropin, and carcinoembryonic antigen) were negative. All these findings are concordant with a DSD with partial gonadal dysgenesis.

**Figure 2:**
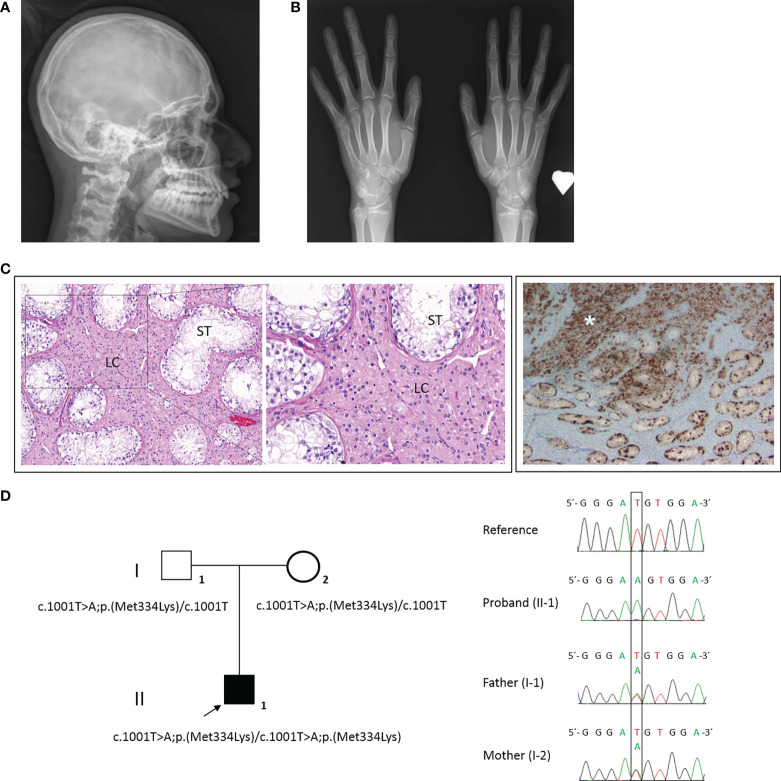
Physical examination, clinical signs, and *HHAT* candidate variant in our 46,XY patient. **(A)** Microcephaly visible in X-rays. **(B)** Skeletal survey showed hypoplasia of distal phalanges of both thumbs and bilateral capito-hamate coalition. **(C)** The anatomopathological analysis of the right and left dysgenetic gonads showed immature seminiferous tubules (ST) with hyperplasia of Leydig cells (LC) (left and magnified image) and a germ-cell (positive for placental alkaline phosphatase (PLAP) staining) tumor in the right gonad (asterisk) (right). **(D)** Family pedigree (right) and electropherogram (left) showing the proband (II-1, arrow) and parental (I-1 and I-2) genomic DNA with a T>A substitution observed in the 1001 position of *HHAT* gene (NM_018194). Both parents were carriers.

The bone age (13 years of chronological age) was determined to be 15 years by the Tanner–Whitehouse method. The skeletal survey at 13.5 years of age revealed microcephaly, with a head circumference of 51.8 cm (SD −2.65) (22) (**Figure 2A**) and hypoplasia of distal phalanges of both thumbs and bilateral capito-hamate coalition (**Figure 2B**). The ophthalmologic evaluation showed an eye fundus with asymmetric papilla (large diameter in right eye), a retinal pigment epithelium atrophy, a temporary flat major excavation in the right eye applied retina, and normal peripheral vision in both eyes.

After 6-month-long psychological counseling, in which the patient confirmed a female gender identity, a laparoscopic gonadectomy was performed. The vaginoscopy showed a vagina terminated in a blind pouch of about 7.5 cm in length, with the absence of the cervix and uterus. Both testes were small, each one measuring around 1.5 × 1 × 0.8 cm. Microscopically, both dysgenetic gonads contained immature seminiferous tubules, 70% of which had germinal cells, and Leydig cell hyperplasia was observed (**Figure 2C**, left and magnified images). In the central zone of the right testis parenchyma, a tumor cell proliferation of 5 mm was identified (**Figure 2C**, right image). Immunohistochemically, the tumor cells were positive for placental alkaline phosphatase (PLAP), cKIT, D2-40, and OCT4 and negative for AFP. No vascular, lymphatic, or spermatic cord invasion was observed. The tumor was classified as a germinoma. No neoplastic infiltration was identified in the left testis. Four months after the gonadectomy, the phallus started to involute, and a feminizing hormonal treatment of 2 µg/day of ethinylestradiol was started.

### Molecular analysis

Genomic DNA was isolated from whole peripheral blood samples from the patient and the parents using standard procedures. DNA samples were analyzed with a custom-designed high-throughput panel with genes related to DSD (DSDSeq.V1, 112 genes and three regulatory regions) using SeqCap EZ technology (Roche Nimblegen) as previously described (23). The genetic result was confirmed by Sanger sequencing. A novel homozygote variant in *HHAT* was identified (MIM: 605743) in the patient, and both parents were carriers. No further variants of interest were identified using the DSDSeq.V1 NGS panel. The *HHAT* (NM_018194) variant c.1001T>A/p.(Met334Lys), located at the end of exon 8, was classified as a variant of uncertain significance (VUS) according to the American College of Medical Genetics and Genomics (ACMG) criteria (PM2 and PP3) (**Figure 2D**). This variant is located close to the exonic end, and possible variant splicing effects were analyzed with the help of Alamut v2.11 splicing tools (SpliceSiteFinder, MaxEntScan, NNSPLICE, GeneSplicer, and Human Splicing Finder), which predicted a possible effect at the nearest splice site.

### Minigene *in vitro* studies

**Figure 3:**
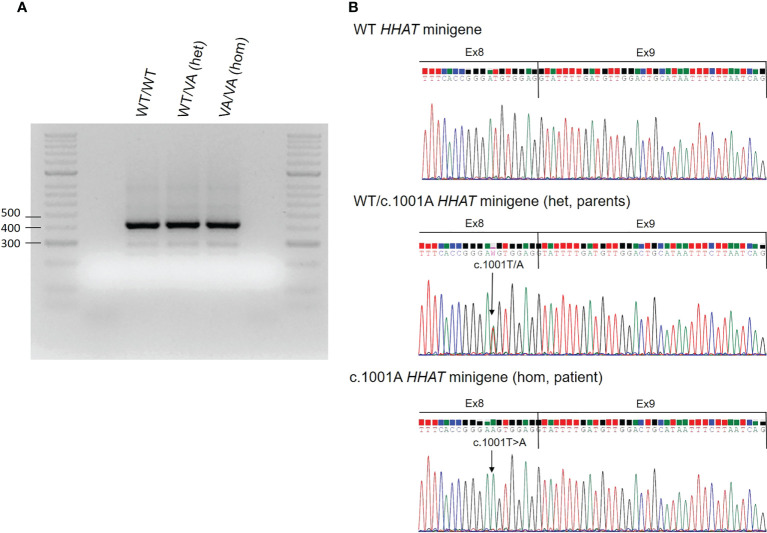
Results of the *HHAT in vitro* minigene functional studies. **(A)** Results of the *HHAT in vitro* minigene experiments. COS-7 cells were transfected with wild-type (WT) HHAT, c.1001A mutant HHAT (MT), or both WT/VA HHAT. RNA was extracted, and RT-PCR for *HHAT* cDNA was performed. The figure shows the PCR products on an agarose gel. **(B)** Electropherograms of the minigene PCR products. The arrow indicates the position of the nucleotide variant c.1001A. Hom, homozygous state; Het, heterozygous state.

RNA sequencing studies were performed with the aim of confirming the *in silico* software prediction that this variant likely affected the nearest splice site. *HHAT* RNA could not be isolated from peripheral blood or saliva, so a minigene *in vitro* study for the *HHAT* variant c.1001T>A was performed. The wild-type (WT) minigene was obtained by PCR from genomic DNA (gDNA) of human whole blood. This minigene was designed to include exons 8 to 10 and ≥200 bp of the flanking introns regions following the Riedmayr et al. protocol (24). The WT PCR-amplified product was cloned into a pcDNA3 vector (Invitrogen by Life Technologies, Carlsbad, CA, USA). The c.1001A mutant minigene (MT) was generated by site-directed mutagenesis following the QuickChange protocol (Agilent Technologies Inc., Santa Clara, CA, USA) and the WT vector as a template. Both vectors were verified by Sanger sequencing. For *in vitro* expression studies, COS-7 cells were grown in a 24-well plate and transiently transfected with the WT, mutant, or both WT and mutant (heterozygous state) minigenes (250 ng/well) using Lipofectamine 2000™ (Invitrogen, Thermo Fisher Scientific, Waltham, MA, USA) and following the manufacturer’s recommendations. Forty-eight hours after transfection, cells were washed with phosphate-buffered saline (PBS) and frozen. RNA was lysed with TRIZOL reagent (Invitrogen, Life Technologies), extracted following an in-house protocol, and treated with DNase (Promega, Madison, WI, USA). cDNA synthesis was performed by reverse transcription (RT)–PCR (GoScript Reverse Transcription System, Promega). Finally, WT and mutant *HHAT* cDNAs were amplified using the cloning primers, loaded in a 2% agarose gel electrophoresis, and sequenced. All minigene transfections (WT/WT, WT/MT, and MT/MT) apparently resulted in cDNA amplification products of the same size, close to the length of minigene design including exons 8 to 10, corresponding to 416 bp (**Figure 3A**). The sequence analysis of these products confirmed that all of them conserved the entire sequence of exons 8, 9, and 10 (**Figure 3B**), which means that the variant does not affect splicing and not confirming the *in silico* prediction of aberrant splicing.

### *In silico* studies

**Figure 4:**
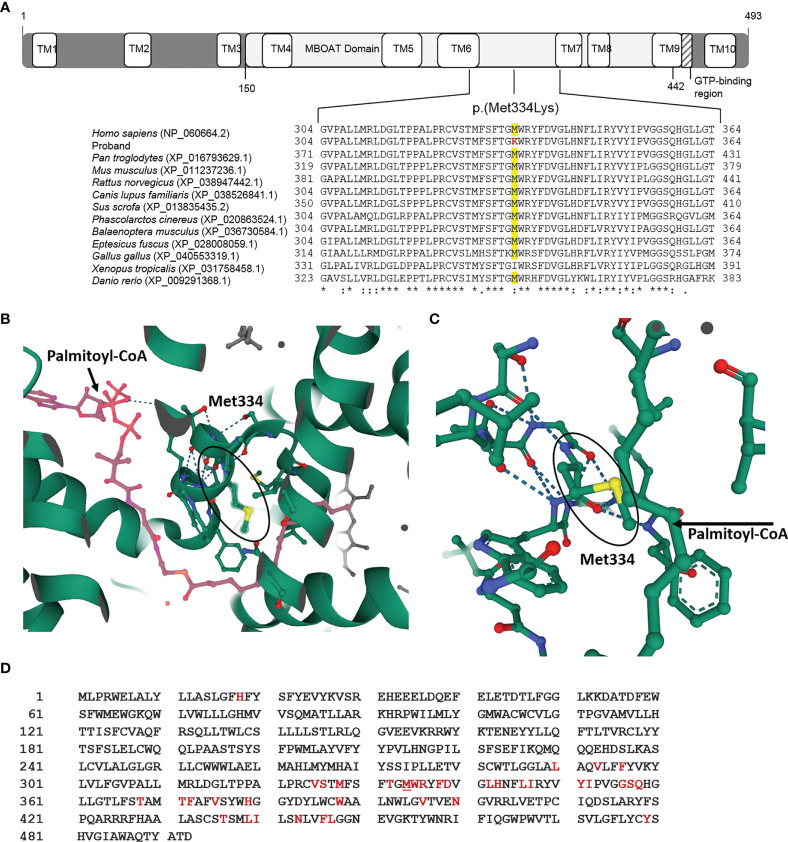
HHAT p.(Met334Lys) variant *in silico* studies. **(A)** Diagram of the HHAT protein isoform NP_060664.2 with its different domains and localization of variant p.(Met334Lys) in membrane-bound *O*-acyltransferase (MBOAT) functional domain of the protein. The *in silico* studies using Clustal Omega showed that the mutated Methionine 334 is conserved through vertebrates. **(B)** 3D image of the HHAT molecule and the ligand palmitoyl-CoA (in pink) indicating the position of Met334 (PBD 7MHY, RCSB PDB). **(C)** 3D magnified image indicating that the 334 position is very close to the ligand palmitoyl-CoA. **(D)** HHAT peptide sequence showing the amino acids in the active center (<8 Å from the ligand) (in red), which includes Met334 (underlined).

As the effect on splicing was not confirmed, this candidate variant was studied *in silico*, analyzing the specific location of the protein change using RCSB PDB (https://www.rcsb.org/), amino acid conservation through evolution by Clustal Omega (https://www.ebi.ac.uk/Tools/msa/clustalo/), and the prediction of protein stability changes caused by the variant using the I-Mutant2.0 website tool (https://folding.biofold.org/i-mutant/i-mutant2.0.html). The online tool HOPE (https://www3.cmbi.umcn.nl/hope/method/) was used for further *in silico* analyses. The *HHAT* variant p.(Met334Lys) is located in the functional MBOAT domain (**Figure 4A**). This domain is important for the main activity of the protein, and a residue change might disturb this function. Regarding amino acid properties, there is a difference in charge, size, and hydrophobicity between the wild-type and the mutant amino acid, because this genetic variant causes a change from a hydrophobic amino acid to a positively charged one. As the mutant amino acid introduces a charge, this can cause the repulsion of ligands or other residues with the same charge. Furthermore, as the mutant residue is bigger, this might lead to bumps. Finally, hydrophobic interactions, either in the core of the protein or on the surface, may be lost. This residue is highly conserved throughout evolution (GERP 5.5599), except for *Xenopus tropicalis*, which presents isoleucine, also a hydrophobic amino acid, in this position. The conservation across species suggests an important role of this amino acid in the HHAT protein structure, stability, or function (**Figure 4A**). Visualizing the HHAT PDB model 7MHY (25), it can be observed that the protein residue Methionine 334 is one of the nearest (5 Å) amino acids to the palmitate donor palmitoyl-CoA (**Figures 4B–D**). Moreover, this single-point variant was predicted to decrease HHAT protein stability, in terms of sequence and protein structure using the same PDB model 7MHY (25).

## Discussion

We describe a case of a homozygous patient for a novel variant c.1001T>A/p.(Met334Lys) in *HHAT* using a custom high-throughput DSD gene panel. *HHAT* gene encodes the hedgehog acyltransferase HHAT, which catalyzes the palmitoylation of the Hh family SHH, DHH, and IHH (1, 8, 9). Therefore, variants in *HHAT* cause a broad range of phenotypes consistent with the defects in Hh pathways (7, 11–19). The patients of the described familial cases with homozygous missense *HHAT* variants also presented with different mixed clinical manifestations (**Figure 1**). Callier et al. (7) described a familial case of two siblings with complex phenotypes including 46,XY DSD, chondrodysplasia, and congenital anomalies (Nivelon–Nivelon–Mabille syndrome). The genetic analysis described a *HHAT* homozygous missense variant (c.860G>T/p.Gly287Val) in the MBOAT domain (**Figure 1**) (7). Abdel-Salam et al. (16) described two sisters of a consanguineous family that presented with microcephaly and cerebellar vermis hypoplasia. One of the sisters presented with brachydactyly with dysplastic nails, and the other one, optic atrophy. There was no evidence of dwarfism or gonadal dysgenesis. An exome sequencing analysis identified a homozygous variant in the MBOAT HHAT domain (c.770T>C/p.Leu257Pro) (**Figure 1**) (16). In a recent publication, Mazen et al. (19) described a patient with 46,XY gonadal dysgenesis, bilateral inguinal testes with no Müllerian derivatives, and strong male identity with microcephaly and multiple café au lait patches. A novel homozygous *HHAT* missense variant (c.1329C>A/p.Asp443Lys) was described as the cause of the patient’s phenotype (**Figure 1**) (19).

In the particular case of the presented patient, although she was firstly referred for DSD with gonadal dysgenesis, extended medical examination confirmed microcephaly with biparietal gliosis, necrosis, and eye abnormalities (**Figure 2**). Considering the role of Hh pathways and their defects, the clinical manifestations of our patient are highly consistent with the alteration of SHH and DHH. The patient also presented with distal phalangeal hypoplasia of both thumbs (**Figure 2B**), which could be closely related to IHH defects. This is concordant with mild skeletal defects caused by heterozygous *IHH* variants in recently published cases (26). However, previously reported *IHH* variants cause more marked defects such as severe short stature, brachydactyly, and severe shortening of the middle phalanges (27, 28). The previously described cases with homozygous variants in *HHAT* showed phenotypes with clinical signs associated with the SHH pathway along with defects in DHH and/or IHH, concordant with our case.

The microscopic analysis of the dysgenetic gonads of our patient showed separated immature seminiferous tubules due to hyperplasia of Leydig cells (**Figure 2C**). Previous studies on *Hhat* loss of function in mice confirmed that Hhat is crucial for proper testis cord formation and differentiation of fetal Leydig cells so that there is a nearly complete absence of Leydig cells in mutant fetal testes (7). In the case of adult mutant testes, a reduced number of Leydig cells compared to normal adult testis with a reminiscent expression of Leydig cell-specific marker CYP11A1 has been described (7). These results are not concordant with ours, as we observed hyperplasia of this cell type in adult testis. Further studies are required to understand the precise function of HHAT in the differentiation of Leydig cells.

The described missense variant in this patient c.1001T>A/p.(Met334Lys) was located at the end of exon 8. Missense variants are usually evaluated focusing on the amino acid change and not on the nucleotide variant, but it has been demonstrated that exonic variants might affect splicing. Based on *in silico* studies, it has been reported that up to 25% of the previously described variants classified as missense and nonsense may affect splicing (29). Therefore, it is highly recommended to use predictive tools for identifying splice variants. In our specific case, the bioinformatics splicing predictor software predicted that the *HHAT* variant c.1001T>A affected the nearest splicing site. For that reason, *in vitro* minigene studies were carried out, which showed that exons 8 to 10 were present in the WT and mutant minigenes (in homozygous and heterozygous states). Therefore, this candidate variant detected in the patient does not affect the splicing, contrary to the prediction (**Figure 3**). Considering these results, we demonstrate the importance of functional studies in order to confirm or refute bioinformatics predictions of variant pathogenicity.

Having concluded that this variant does not affect RNA splicing, we decided to perform *in silico* studies to analyze its pathogenicity. The variant is located at the end of exon 8, causing the peptide sequence to change from Methionine to Lysine in 334 positions. It is located in the highly conserved MBOAT functional domain (**Figure 4**). From the previously described variants in *HHAT*, those that cause a phenotype similar to that of our patient are also located in MBOAT. The affected amino acid is highly conserved through evolution (**Figure 4**) and is near the ligand palmitoyl-CoA, and the variant seems to affect HHAT protein stability. All these results lead us to think that the variant does affect the protein function and, therefore, might be the cause of the patient’s phenotype. However, as genetic variants in *HHAT* cause a broad spectrum of phenotypes and high variability in their severity, it would be interesting to conduct functional studies to confirm the cell downstream signaling defects resulting from genetic variants in *HHAT* and improve understanding of Hh pathways.

In conclusion, we described a novel *HHAT* variant in a patient with DSD with partial gonadal dysgenesis, microcephaly, eye defects, and hypoplasia of the distal phalanges of both thumbs. This candidate variant was initially predicted to affect splicing, but *in vitro* studies refuted this. As the *in silico* studies supported the pathogenicity of the variant, we considered it to be the cause of the patient’s phenotype.

## Data availability statement

The datasets for this article are not publicly available due to concerns regarding participant/patient anonymity. Requests to access the datasets should be directed to the corresponding author.

## Ethics statement

The studies involving human participants were reviewed and approved by CEIC Hospital Universitari Vall d’Hebron. Written informed consent to participate in this study was provided by the participants’ legal guardian/next of kin.Written informed consent was obtained from the minor(s)’ legal guardian/next of kin for the publication of any potentially identifiable images or data included in this article.

## Author contributions

NB-R and LS-C wrote the manuscript. NB-R and NC-T contributed to the conception and design of the study, and performed experimental studies. MF-C and SB-S performed genetic studies. MG and TM performed the histological analyses. DY, MC and MG contributed to the diagnosis of the patient. All authors contributed to manuscript revision, read, and approved the submitted version. DY and NC-T supervised the project.

## Funding

This study was partly supported by a grant from the Fondo de Investigación Sanitaria (PI15/01647 [to MF-C and SB-S]).

## Acknowledgments

We thank Dr. Josep Castellví for his help and expertise and Dr. Javier Hernández (Tumor Tissue Bank, Biobanc HUVH) for the availability of the samples.

## Conflict of interest

The authors declare that the research was conducted in the absence of any commercial or financial relationships that could be construed as a potential conflict of interest.

## Publisher’s note

All claims expressed in this article are solely those of the authors and do not necessarily represent those of their affiliated organizations, or those of the publisher, the editors and the reviewers. Any product that may be evaluated in this article, or claim that may be made by its manufacturer, is not guaranteed or endorsed by the publisher.
